# 1,2-Bis{2-[(1,3-benzothia­zol-2-yl)sulfanylmeth­yl]phen­oxy}ethane

**DOI:** 10.1107/S160053681003062X

**Published:** 2010-08-11

**Authors:** Liang-Wei Zhang, Wen-Yu Wu, Zhong-Xing Su, Ai-Jiang Zhang, Xiang Liu

**Affiliations:** a State Key Laboratory of Applied Organic Chemistry, College of Chemistry and Chemical Engineering, Lanzhou University, Lanzhou 730000, People’s Republic of China

## Abstract

The mol­ecule of the title compound, C_30_H_24_N_2_O_2_S_4_, adopts a Z-shaped conformation. The terminal benzothia­zole ring systems are oriented at a dihedral angle of 60.81 (8)°, while the central benzene rings are twisted to each other by a dihedral angle of 13.56 (14)°. Weak inter­molecular C—H⋯π inter­actions are present in the crystal structure.

## Related literature

For the biological activity of benzothia­zoles and their deriva­tives, see: Paramashivappa *et al.* (2002 ▶); Kočí *et al.* (2002 ▶); Fei *et al.* (2009 ▶). For the preparation of the title compound, see: Yuan *et al.* (2005 ▶); Siva & Murugan (2005 ▶); Gruter *et al.* (1994 ▶); Kumar *et al.* (2005 ▶).
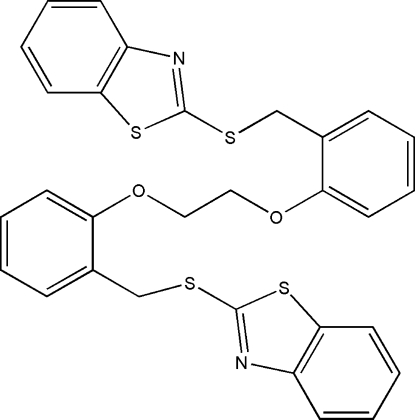


## Experimental

### 

#### Crystal data

C_30_H_24_N_2_O_2_S_4_
*M* *_r_* = 572.75
Triclinic, 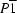

*a* = 9.8194 (6) Å
*b* = 10.7740 (8) Å
*c* = 14.0716 (9) Å
α = 82.422 (1)°
β = 76.285 (1)°
γ = 68.993 (1)°
*V* = 1348.32 (16) Å^3^
*Z* = 2
Mo *K*α radiation
μ = 0.39 mm^−1^
*T* = 296 K
0.35 × 0.32 × 0.30 mm

#### Data collection

Bruker APEXII CCD diffractometer
Absorption correction: multi-scan (*SADABS*; Bruker, 2001 ▶) *T*_min_ = 0.877, *T*_max_ = 0.893
7093 measured reflections
4947 independent reflections
3531 reflections with *I* > 2σ(*I*)
*R*_int_ = 0.022

#### Refinement

*R*[*F* ^2^ > 2σ(*F* ^2^)] = 0.044
*wR*(*F* ^2^) = 0.101
*S* = 1.01
4947 reflections
343 parameters
H-atom parameters constrained
Δρ_max_ = 0.24 e Å^−3^
Δρ_min_ = −0.28 e Å^−3^

### 

Data collection: *APEX2* (Bruker, 2007 ▶); cell refinement: *SAINT* (Bruker, 2007 ▶); data reduction: *SAINT*; program(s) used to solve structure: *SHELXS97* (Sheldrick, 2008 ▶); program(s) used to refine structure: *SHELXL97* (Sheldrick, 2008 ▶); molecular graphics: *SHELXTL* (Sheldrick, 2008 ▶); software used to prepare material for publication: *SHELXTL*.

## Supplementary Material

Crystal structure: contains datablocks I, global. DOI: 10.1107/S160053681003062X/xu5002sup1.cif

Structure factors: contains datablocks I. DOI: 10.1107/S160053681003062X/xu5002Isup2.hkl

Additional supplementary materials:  crystallographic information; 3D view; checkCIF report

## Figures and Tables

**Table 1:** Hydrogen-bond geometry (Å, °) *Cg*2, *Cg*3,and *Cg*5 are centroids of the S4,C24,N2,C25,C30, C1–C6 and C17–C22 rings, respectively.

*D*—H⋯*A*	*D*—H	H⋯*A*	*D*⋯*A*	*D*—H⋯*A*
C3—H3⋯*Cg*2^i^	0.93	2.91	3.727 (3)	147
C13—H13⋯*Cg*3^ii^	0.93	2.87	3.705 (3)	149
C18—H18⋯*Cg*3	0.93	2.81	3.636 (3)	149
C21—H21⋯*Cg*2^iii^	0.93	2.78	3.457 (3)	131
C29—H29⋯*Cg*5^ii^	0.93	2.79	3.559 (3)	141

Symmetry codes: (i) 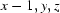; (ii) 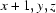; (iii) 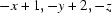.

**Fig. 1.**
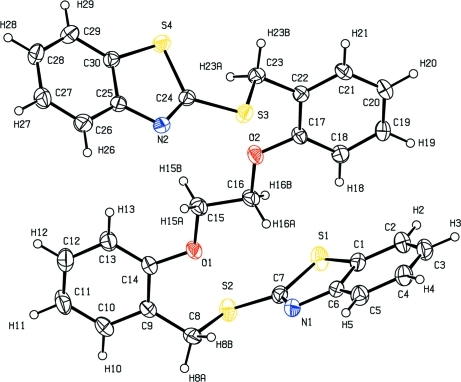
The molecular structure of the title compound. Displacement ellipsoids are drawn at the 30% probability level.

**Fig. 2.**
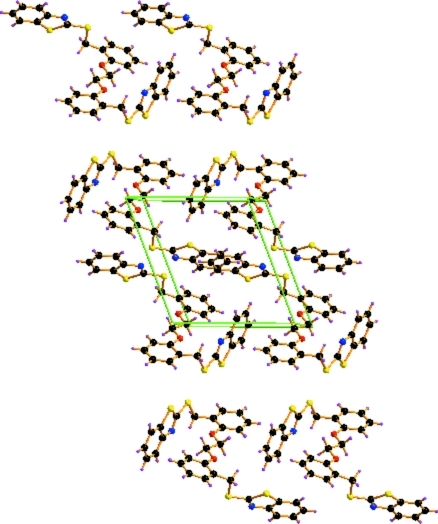
Molecular packing of the title compound with the weak intermolecular S···S and C—H···π interactions.

## supplementary crystallographic information

### Comment

It is reported that benzothiazoles and their derivatives have showed a wide variety of biological activities, such as anti-inflammatory, antimycobacteriva, and capability of labeling cancer cells (Paramashivappa *et al.*, 2002; Kočí *et al.*, 2002; Fei *et al.*, 2009). As an important class of benzothiazoles, benzothiazole-2-thiol also exhibits potential biological activities. Therefore, the title compound was synthesized.

In the title compound (Fig. 1), the dihedral angles between each benzene ring and benzothiazole ring pair where the two rings are linked to each other are 87.00and 60.18°, respectively, while the dihedral angle between the two benzene rings is 13.63°. The stability of the structure is ascribed to the weak C—H···π interactions. In the crystal (Fig. 2), the molecules are linked by weak C—H···π interactions and intermolecular S···S interactions.

### Experimental

All reagents and solvents were obtained from commercial sources and needed to be further purified. 1,2-bis(*o*-tolyloxy)ethane was synthesized according to the method proposed by Yuan *et al.* (2005). 1,2-Bis(2-(bromomethyl)phenoxy)ethane was prepared following previously reported methods, but with some modification (Siva & Murugan, 2005; Gruter *et al.*, 1994). 1,2-Bis-(*o*-tolyloxy)ethane (10 mmol), *N*-bromosuccinamide (30 mmol), benzoyl peroxide (20 mmol) and CCl_4_ (50 ml) were taken in a 100 ml round-bottom flask. The reaction mixture was refluxed for 6 h under an IR lamp (250 W) irradiation. Subsequently, the solid formed in the reaction was removed by fltration. The solvent was evaporated under vacuum to give the crude product, which was thereafter recrystallized (ethanol-acetone) to prodcue pale-yellow needles, yield: 76%. The title compound was synthesized according to the related literature (Kumar *et al.*, 2005). A mixture of 2-mercaptobenzothiazole (4 mmol), 1,2-bis(2-(bromomethyl)*p*-henoxy)ethane (2 mmol) and finely grounded anhydrous K_2_CO_3_ (4 mmol) in acetone was refluxed for 2 h (TLC monitoring). The reaction mixture was then cooled to room temperature and filtered. Evaporation of the filtrate yielded the crude products. Finally, the crude products were purified by recrystallization from the hexane-ethyl acetate solution, yield: 84%.

### Refinement

H atoms were placed in calculated positions and were refined in a riding-model approximation with C—H = 0.93 or 0.97 Å and with *U*_iso_(H) = 1.2*U*_eq_(C).

### Crystal data

**Table:**

C_30_H_24_N_2_O_2_S_4_	*Z* = 2
*M**_r_* = 572.75	*F*(000) = 596
Triclinic, *P*1	*D*_x_ = 1.411 Mg m^−^^3^
Hall symbol: -P 1	Mo *K*α radiation, λ = 0.71073 Å
*a* = 9.8194 (6) Å	Cell parameters from 1819 reflections
*b* = 10.7740 (8) Å	θ = 2.3–22.8°
*c* = 14.0716 (9) Å	µ = 0.39 mm^−^^1^
α = 82.422 (1)°	*T* = 296 K
β = 76.285 (1)°	Block, yellow
γ = 68.993 (1)°	0.35 × 0.32 × 0.30 mm
*V* = 1348.32 (16) Å^3^	

### Data collection

**Table:**

Bruker APEXII CCD diffractometer	4947 independent reflections
Radiation source: fine-focus sealed tube	3531 reflections with *I* > 2σ(*I*)
graphite	*R*_int_ = 0.022
φ and ω scans	θ_max_ = 25.5°, θ_min_ = 1.5°
Absorption correction: multi-scan (*SADABS*; Bruker, 2001)	*h* = −11→10
*T*_min_ = 0.877, *T*_max_ = 0.893	*k* = −11→13
7093 measured reflections	*l* = −17→10

### Refinement

**Table:**

Refinement on *F*^2^	Primary atom site location: structure-invariant direct methods
Least-squares matrix: full	Secondary atom site location: difference Fourier map
*R*[*F*^2^ > 2σ(*F*^2^)] = 0.044	Hydrogen site location: inferred from neighbouring sites
*wR*(*F*^2^) = 0.101	H-atom parameters constrained
*S* = 1.01	*w* = 1/[σ^2^(*F*_o_^2^) + (0.0313*P*)^2^ + 0.6717*P*] where *P* = (*F*_o_^2^ + 2*F*_c_^2^)/3
4947 reflections	(Δ/σ)_max_ < 0.001
343 parameters	Δρ_max_ = 0.24 e Å^−^^3^
0 restraints	Δρ_min_ = −0.28 e Å^−^^3^

### Special details

**Table:**

Geometry. All e.s.d.'s (except the e.s.d. in the dihedral angle between two l.s. planes) are estimated using the full covariance matrix. The cell e.s.d.'s are taken into account individually in the estimation of e.s.d.'s in distances, angles and torsion angles; correlations between e.s.d.'s in cell parameters are only used when they are defined by crystal symmetry. An approximate (isotropic) treatment of cell e.s.d.'s is used for estimating e.s.d.'s involving l.s. planes.
Refinement. Refinement of *F*^2^ against ALL reflections. The weighted *R*-factor *wR* and goodness of fit *S* are based on *F*^2^, conventional *R*-factors *R* are based on *F*, with *F* set to zero for negative *F*^2^. The threshold expression of *F*^2^ > σ(*F*^2^) is used only for calculating *R*-factors(gt) *etc*. and is not relevant to the choice of reflections for refinement. *R*-factors based on *F*^2^ are statistically about twice as large as those based on *F*, and *R*- factors based on ALL data will be even larger.

### Fractional atomic coordinates and isotropic or equivalent isotropic displacement parameters (Å^2^)

**Table:**

	*x*	*y*	*z*	*U*_iso_*/*U*_eq_	
S1	0.25411 (9)	0.18208 (8)	0.27373 (6)	0.0552 (2)	
S2	0.38885 (9)	0.13160 (8)	0.44913 (6)	0.0523 (2)	
S3	0.47855 (8)	0.88320 (7)	0.11847 (5)	0.0451 (2)	
S4	0.80655 (8)	0.86358 (8)	0.05966 (5)	0.0463 (2)	
N1	0.1731 (2)	0.3580 (2)	0.40420 (16)	0.0432 (6)	
N2	0.6259 (2)	0.9606 (2)	0.22042 (15)	0.0402 (5)	
O1	0.5007 (2)	0.38268 (19)	0.38482 (13)	0.0479 (5)	
O2	0.4699 (2)	0.58394 (19)	0.16478 (13)	0.0474 (5)	
C1	0.0873 (3)	0.4137 (3)	0.33328 (18)	0.0387 (6)	
C2	−0.0182 (3)	0.5403 (3)	0.3337 (2)	0.0503 (7)	
H2	−0.0354	0.5968	0.3831	0.060*	
C3	−0.0972 (3)	0.5814 (3)	0.2596 (2)	0.0531 (8)	
H3	−0.1679	0.6664	0.2591	0.064*	
C4	−0.0727 (3)	0.4980 (3)	0.1859 (2)	0.0536 (8)	
H4	−0.1282	0.5274	0.1372	0.064*	
C5	0.0320 (3)	0.3725 (3)	0.1834 (2)	0.0512 (8)	
H5	0.0485	0.3168	0.1336	0.061*	
C6	0.1124 (3)	0.3313 (3)	0.2573 (2)	0.0413 (6)	
C7	0.2632 (3)	0.2401 (3)	0.38187 (19)	0.0407 (6)	
C8	0.3907 (3)	0.2396 (3)	0.53801 (19)	0.0484 (7)	
H8A	0.3180	0.3272	0.5299	0.058*	
H8B	0.3612	0.2041	0.6036	0.058*	
C9	0.5399 (3)	0.2527 (3)	0.52779 (18)	0.0396 (6)	
C10	0.6279 (3)	0.1936 (3)	0.5960 (2)	0.0530 (8)	
H10	0.5951	0.1419	0.6484	0.064*	
C11	0.7635 (4)	0.2098 (4)	0.5880 (2)	0.0670 (10)	
H11	0.8214	0.1688	0.6344	0.080*	
C12	0.8124 (4)	0.2859 (4)	0.5117 (2)	0.0664 (9)	
H12	0.9034	0.2973	0.5068	0.080*	
C13	0.7282 (3)	0.3463 (3)	0.4420 (2)	0.0520 (8)	
H13	0.7623	0.3978	0.3900	0.062*	
C14	0.5929 (3)	0.3296 (3)	0.44972 (19)	0.0399 (6)	
C15	0.5447 (3)	0.4641 (3)	0.30440 (19)	0.0445 (7)	
H15A	0.6418	0.4161	0.2664	0.053*	
H15B	0.5490	0.5437	0.3270	0.053*	
C16	0.4271 (3)	0.4995 (3)	0.24431 (18)	0.0432 (7)	
H16A	0.4244	0.4202	0.2204	0.052*	
H16B	0.3297	0.5458	0.2827	0.052*	
C17	0.3904 (3)	0.6233 (2)	0.09199 (18)	0.0364 (6)	
C18	0.2737 (3)	0.5827 (3)	0.0873 (2)	0.0430 (7)	
H18	0.2423	0.5267	0.1367	0.052*	
C19	0.2042 (3)	0.6262 (3)	0.0083 (2)	0.0453 (7)	
H19	0.1255	0.5993	0.0051	0.054*	
C20	0.2497 (3)	0.7087 (3)	−0.0653 (2)	0.0459 (7)	
H20	0.2030	0.7366	−0.1185	0.055*	
C21	0.3653 (3)	0.7495 (3)	−0.05950 (18)	0.0411 (7)	
H21	0.3958	0.8056	−0.1093	0.049*	
C22	0.4369 (3)	0.7090 (2)	0.01829 (18)	0.0353 (6)	
C23	0.5585 (3)	0.7579 (3)	0.02708 (18)	0.0398 (6)	
H23A	0.5961	0.7963	−0.0354	0.048*	
H23B	0.6403	0.6848	0.0472	0.048*	
C24	0.6343 (3)	0.9053 (3)	0.14192 (18)	0.0363 (6)	
C25	0.7615 (3)	0.9749 (3)	0.21972 (18)	0.0371 (6)	
C26	0.7899 (3)	1.0335 (3)	0.2921 (2)	0.0515 (8)	
H26	0.7157	1.0665	0.3465	0.062*	
C27	0.9282 (3)	1.0417 (3)	0.2820 (2)	0.0547 (8)	
H27	0.9481	1.0807	0.3299	0.066*	
C28	1.0395 (3)	0.9924 (3)	0.2009 (2)	0.0513 (8)	
H28	1.1328	0.9994	0.1954	0.062*	
C29	1.0153 (3)	0.9342 (3)	0.1293 (2)	0.0474 (7)	
H29	1.0908	0.9005	0.0757	0.057*	
C30	0.8741 (3)	0.9266 (3)	0.13876 (18)	0.0374 (6)	

### Atomic displacement parameters (Å^2^)

**Table:**

	*U*^11^	*U*^22^	*U*^33^	*U*^12^	*U*^13^	*U*^23^
S1	0.0584 (5)	0.0437 (4)	0.0664 (5)	−0.0079 (4)	−0.0286 (4)	−0.0108 (4)
S2	0.0542 (5)	0.0422 (4)	0.0638 (5)	−0.0135 (4)	−0.0279 (4)	0.0061 (4)
S3	0.0378 (4)	0.0500 (4)	0.0513 (4)	−0.0185 (3)	−0.0077 (3)	−0.0086 (3)
S4	0.0419 (4)	0.0579 (5)	0.0430 (4)	−0.0221 (4)	−0.0020 (3)	−0.0130 (3)
N1	0.0376 (13)	0.0486 (15)	0.0428 (13)	−0.0121 (11)	−0.0114 (11)	−0.0018 (11)
N2	0.0387 (13)	0.0478 (14)	0.0362 (13)	−0.0192 (11)	−0.0053 (10)	−0.0009 (10)
O1	0.0507 (12)	0.0590 (13)	0.0407 (11)	−0.0260 (10)	−0.0219 (9)	0.0186 (9)
O2	0.0552 (12)	0.0556 (12)	0.0443 (11)	−0.0319 (10)	−0.0253 (10)	0.0184 (9)
C1	0.0325 (14)	0.0469 (17)	0.0384 (15)	−0.0156 (13)	−0.0097 (12)	0.0025 (13)
C2	0.0449 (17)	0.0493 (18)	0.0533 (18)	−0.0079 (14)	−0.0136 (14)	−0.0082 (14)
C3	0.0460 (18)	0.0505 (19)	0.058 (2)	−0.0080 (15)	−0.0185 (15)	0.0025 (15)
C4	0.0505 (19)	0.059 (2)	0.0560 (19)	−0.0182 (16)	−0.0266 (15)	0.0095 (16)
C5	0.0550 (19)	0.056 (2)	0.0511 (18)	−0.0213 (16)	−0.0223 (15)	−0.0051 (15)
C6	0.0382 (16)	0.0431 (16)	0.0465 (16)	−0.0160 (13)	−0.0135 (13)	−0.0004 (13)
C7	0.0375 (16)	0.0430 (17)	0.0457 (16)	−0.0182 (13)	−0.0125 (13)	0.0039 (13)
C8	0.0484 (18)	0.0574 (19)	0.0344 (15)	−0.0146 (15)	−0.0087 (13)	0.0060 (13)
C9	0.0444 (16)	0.0392 (16)	0.0332 (14)	−0.0095 (13)	−0.0117 (12)	−0.0010 (12)
C10	0.063 (2)	0.0532 (19)	0.0415 (17)	−0.0156 (16)	−0.0222 (15)	0.0095 (14)
C11	0.069 (2)	0.076 (2)	0.065 (2)	−0.022 (2)	−0.0438 (19)	0.0129 (19)
C12	0.059 (2)	0.085 (3)	0.069 (2)	−0.0321 (19)	−0.0317 (18)	0.006 (2)
C13	0.0577 (19)	0.060 (2)	0.0500 (18)	−0.0305 (16)	−0.0222 (15)	0.0093 (15)
C14	0.0438 (16)	0.0391 (16)	0.0381 (15)	−0.0115 (13)	−0.0159 (13)	0.0000 (12)
C15	0.0535 (18)	0.0451 (17)	0.0400 (16)	−0.0223 (14)	−0.0165 (13)	0.0092 (13)
C16	0.0516 (17)	0.0456 (17)	0.0371 (15)	−0.0234 (14)	−0.0147 (13)	0.0117 (12)
C17	0.0430 (16)	0.0354 (15)	0.0341 (14)	−0.0144 (12)	−0.0152 (12)	0.0037 (11)
C18	0.0489 (17)	0.0422 (17)	0.0442 (16)	−0.0230 (14)	−0.0141 (13)	0.0062 (13)
C19	0.0438 (17)	0.0482 (17)	0.0527 (18)	−0.0187 (14)	−0.0195 (14)	−0.0060 (14)
C20	0.0550 (18)	0.0471 (17)	0.0395 (16)	−0.0140 (15)	−0.0224 (14)	−0.0020 (13)
C21	0.0477 (17)	0.0403 (16)	0.0320 (14)	−0.0105 (13)	−0.0106 (12)	0.0016 (12)
C22	0.0366 (15)	0.0330 (14)	0.0352 (14)	−0.0103 (12)	−0.0081 (11)	−0.0017 (11)
C23	0.0407 (16)	0.0417 (16)	0.0389 (15)	−0.0186 (13)	−0.0063 (12)	0.0009 (12)
C24	0.0362 (15)	0.0344 (15)	0.0382 (15)	−0.0143 (12)	−0.0076 (12)	0.0051 (12)
C25	0.0364 (15)	0.0387 (15)	0.0361 (15)	−0.0145 (12)	−0.0080 (12)	0.0039 (12)
C26	0.0506 (19)	0.063 (2)	0.0437 (17)	−0.0228 (16)	−0.0056 (14)	−0.0106 (15)
C27	0.055 (2)	0.065 (2)	0.0519 (19)	−0.0229 (17)	−0.0191 (16)	−0.0102 (16)
C28	0.0379 (17)	0.059 (2)	0.063 (2)	−0.0198 (15)	−0.0142 (15)	−0.0084 (16)
C29	0.0374 (16)	0.0505 (18)	0.0521 (18)	−0.0150 (14)	−0.0030 (13)	−0.0061 (14)
C30	0.0369 (15)	0.0370 (15)	0.0389 (15)	−0.0140 (12)	−0.0082 (12)	0.0002 (12)

### Geometric parameters (Å, °)

**Table:**

S1—C6	1.739 (3)	C11—H11	0.9300
S1—C7	1.752 (3)	C12—C13	1.379 (4)
S2—C7	1.736 (3)	C12—H12	0.9300
S2—C8	1.824 (3)	C13—C14	1.381 (4)
S3—C24	1.740 (3)	C13—H13	0.9300
S3—C23	1.820 (3)	C15—C16	1.501 (3)
S4—C30	1.736 (3)	C15—H15A	0.9700
S4—C24	1.751 (3)	C15—H15B	0.9700
N1—C7	1.290 (3)	C16—H16A	0.9700
N1—C1	1.394 (3)	C16—H16B	0.9700
N2—C24	1.295 (3)	C17—C18	1.383 (3)
N2—C25	1.393 (3)	C17—C22	1.400 (3)
O1—C14	1.365 (3)	C18—C19	1.383 (4)
O1—C15	1.419 (3)	C18—H18	0.9300
O2—C17	1.366 (3)	C19—C20	1.372 (4)
O2—C16	1.428 (3)	C19—H19	0.9300
C1—C2	1.386 (4)	C20—C21	1.379 (4)
C1—C6	1.400 (4)	C20—H20	0.9300
C2—C3	1.378 (4)	C21—C22	1.379 (3)
C2—H2	0.9300	C21—H21	0.9300
C3—C4	1.383 (4)	C22—C23	1.502 (3)
C3—H3	0.9300	C23—H23A	0.9700
C4—C5	1.373 (4)	C23—H23B	0.9700
C4—H4	0.9300	C25—C30	1.392 (3)
C5—C6	1.385 (4)	C25—C26	1.394 (4)
C5—H5	0.9300	C26—C27	1.365 (4)
C8—C9	1.494 (4)	C26—H26	0.9300
C8—H8A	0.9700	C27—C28	1.390 (4)
C8—H8B	0.9700	C27—H27	0.9300
C9—C10	1.383 (4)	C28—C29	1.361 (4)
C9—C14	1.401 (4)	C28—H28	0.9300
C10—C11	1.381 (4)	C29—C30	1.392 (4)
C10—H10	0.9300	C29—H29	0.9300
C11—C12	1.364 (4)		
			
C6—S1—C7	88.75 (13)	C16—C15—H15A	110.7
C7—S2—C8	102.31 (13)	O1—C15—H15B	110.7
C24—S3—C23	103.19 (12)	C16—C15—H15B	110.7
C30—S4—C24	88.64 (12)	H15A—C15—H15B	108.8
C7—N1—C1	110.3 (2)	O2—C16—C15	104.9 (2)
C24—N2—C25	110.1 (2)	O2—C16—H16A	110.8
C14—O1—C15	117.9 (2)	C15—C16—H16A	110.8
C17—O2—C16	118.58 (19)	O2—C16—H16B	110.8
C2—C1—N1	125.3 (2)	C15—C16—H16B	110.8
C2—C1—C6	119.4 (2)	H16A—C16—H16B	108.8
N1—C1—C6	115.3 (2)	O2—C17—C18	124.5 (2)
C3—C2—C1	119.0 (3)	O2—C17—C22	115.0 (2)
C3—C2—H2	120.5	C18—C17—C22	120.5 (2)
C1—C2—H2	120.5	C17—C18—C19	119.3 (2)
C2—C3—C4	120.9 (3)	C17—C18—H18	120.3
C2—C3—H3	119.6	C19—C18—H18	120.3
C4—C3—H3	119.6	C20—C19—C18	121.0 (3)
C5—C4—C3	121.3 (3)	C20—C19—H19	119.5
C5—C4—H4	119.4	C18—C19—H19	119.5
C3—C4—H4	119.4	C19—C20—C21	119.2 (2)
C4—C5—C6	118.0 (3)	C19—C20—H20	120.4
C4—C5—H5	121.0	C21—C20—H20	120.4
C6—C5—H5	121.0	C20—C21—C22	121.6 (3)
C5—C6—C1	121.5 (3)	C20—C21—H21	119.2
C5—C6—S1	129.4 (2)	C22—C21—H21	119.2
C1—C6—S1	109.18 (19)	C21—C22—C17	118.3 (2)
N1—C7—S2	126.9 (2)	C21—C22—C23	121.8 (2)
N1—C7—S1	116.4 (2)	C17—C22—C23	119.8 (2)
S2—C7—S1	116.72 (16)	C22—C23—S3	107.34 (17)
C9—C8—S2	112.85 (19)	C22—C23—H23A	110.2
C9—C8—H8A	109.0	S3—C23—H23A	110.2
S2—C8—H8A	109.0	C22—C23—H23B	110.2
C9—C8—H8B	109.0	S3—C23—H23B	110.2
S2—C8—H8B	109.0	H23A—C23—H23B	108.5
H8A—C8—H8B	107.8	N2—C24—S3	120.79 (19)
C10—C9—C14	117.8 (3)	N2—C24—S4	116.25 (19)
C10—C9—C8	121.6 (3)	S3—C24—S4	122.87 (15)
C14—C9—C8	120.6 (2)	N2—C25—C30	115.4 (2)
C11—C10—C9	121.3 (3)	N2—C25—C26	125.1 (2)
C11—C10—H10	119.3	C30—C25—C26	119.5 (2)
C9—C10—H10	119.3	C27—C26—C25	119.1 (3)
C12—C11—C10	119.8 (3)	C27—C26—H26	120.5
C12—C11—H11	120.1	C25—C26—H26	120.5
C10—C11—H11	120.1	C26—C27—C28	120.7 (3)
C11—C12—C13	120.7 (3)	C26—C27—H27	119.7
C11—C12—H12	119.7	C28—C27—H27	119.7
C13—C12—H12	119.7	C29—C28—C27	121.6 (3)
C14—C13—C12	119.5 (3)	C29—C28—H28	119.2
C14—C13—H13	120.2	C27—C28—H28	119.2
C12—C13—H13	120.2	C28—C29—C30	118.0 (3)
O1—C14—C13	125.0 (2)	C28—C29—H29	121.0
O1—C14—C9	114.2 (2)	C30—C29—H29	121.0
C13—C14—C9	120.8 (2)	C29—C30—C25	121.2 (2)
O1—C15—C16	105.2 (2)	C29—C30—S4	129.3 (2)
O1—C15—H15A	110.7	C25—C30—S4	109.55 (19)
			
C7—N1—C1—C2	−178.5 (3)	O1—C15—C16—O2	178.7 (2)
C7—N1—C1—C6	2.0 (3)	C16—O2—C17—C18	−2.8 (4)
N1—C1—C2—C3	−178.7 (3)	C16—O2—C17—C22	178.2 (2)
C6—C1—C2—C3	0.8 (4)	O2—C17—C18—C19	−178.1 (2)
C1—C2—C3—C4	0.3 (4)	C22—C17—C18—C19	0.8 (4)
C2—C3—C4—C5	−0.8 (5)	C17—C18—C19—C20	0.2 (4)
C3—C4—C5—C6	0.3 (5)	C18—C19—C20—C21	−0.7 (4)
C4—C5—C6—C1	0.8 (4)	C19—C20—C21—C22	0.3 (4)
C4—C5—C6—S1	−178.3 (2)	C20—C21—C22—C17	0.7 (4)
C2—C1—C6—C5	−1.4 (4)	C20—C21—C22—C23	−177.3 (2)
N1—C1—C6—C5	178.2 (2)	O2—C17—C22—C21	177.8 (2)
C2—C1—C6—S1	178.0 (2)	C18—C17—C22—C21	−1.2 (4)
N1—C1—C6—S1	−2.5 (3)	O2—C17—C22—C23	−4.2 (3)
C7—S1—C6—C5	−179.0 (3)	C18—C17—C22—C23	176.8 (2)
C7—S1—C6—C1	1.8 (2)	C21—C22—C23—S3	103.3 (2)
C1—N1—C7—S2	−178.76 (19)	C17—C22—C23—S3	−74.7 (3)
C1—N1—C7—S1	−0.5 (3)	C24—S3—C23—C22	167.45 (18)
C8—S2—C7—N1	−12.9 (3)	C25—N2—C24—S3	−176.17 (18)
C8—S2—C7—S1	168.86 (15)	C25—N2—C24—S4	0.5 (3)
C6—S1—C7—N1	−0.8 (2)	C23—S3—C24—N2	−162.0 (2)
C6—S1—C7—S2	177.66 (16)	C23—S3—C24—S4	21.6 (2)
C7—S2—C8—C9	−117.2 (2)	C30—S4—C24—N2	0.1 (2)
S2—C8—C9—C10	−108.3 (3)	C30—S4—C24—S3	176.65 (18)
S2—C8—C9—C14	73.8 (3)	C24—N2—C25—C30	−1.0 (3)
C14—C9—C10—C11	0.2 (4)	C24—N2—C25—C26	178.4 (3)
C8—C9—C10—C11	−177.8 (3)	N2—C25—C26—C27	−179.7 (3)
C9—C10—C11—C12	0.3 (5)	C30—C25—C26—C27	−0.3 (4)
C10—C11—C12—C13	−0.5 (5)	C25—C26—C27—C28	0.0 (5)
C11—C12—C13—C14	0.2 (5)	C26—C27—C28—C29	−0.2 (5)
C15—O1—C14—C13	−1.7 (4)	C27—C28—C29—C30	0.8 (5)
C15—O1—C14—C9	178.8 (2)	C28—C29—C30—C25	−1.1 (4)
C12—C13—C14—O1	−179.1 (3)	C28—C29—C30—S4	178.0 (2)
C12—C13—C14—C9	0.3 (4)	N2—C25—C30—C29	−179.7 (2)
C10—C9—C14—O1	179.0 (2)	C26—C25—C30—C29	0.9 (4)
C8—C9—C14—O1	−3.0 (4)	N2—C25—C30—S4	1.0 (3)
C10—C9—C14—C13	−0.5 (4)	C26—C25—C30—S4	−178.4 (2)
C8—C9—C14—C13	177.5 (3)	C24—S4—C30—C29	−179.8 (3)
C14—O1—C15—C16	176.6 (2)	C24—S4—C30—C25	−0.6 (2)
C17—O2—C16—C15	174.3 (2)		

### Hydrogen-bond geometry (°)

**Table:**

Cg2, Cg3,and Cg5 are centroids of the S4,C24,N2,C25,C30, C1–C6 and C17–C22 rings, respectively.

**Table:**

*D*—H···*A*
—···
—···
—···
—···
—···
